# Programmed cell death-ligand 2: new insights in cancer

**DOI:** 10.3389/fimmu.2024.1359532

**Published:** 2024-03-28

**Authors:** Yukang Yang, Xia Yan, Xueqi Bai, Jiayang Yang, Jianbo Song

**Affiliations:** ^1^ Third Hospital of Shanxi Medical University, Shanxi Bethune Hospital, Shanxi Academy of Medical Sciences Tongji Shanxi Hospital, Taiyuan, China; ^2^ Cancer Center, Shanxi Bethune Hospital, Shanxi Academy of Medical Sciences, Tongji Shanxi Hospital, Third Hospital of Shanxi Medical University, Taiyuan, China

**Keywords:** PD-1, PD-L2, cancer, immunity, combination therapy

## Abstract

Immunotherapy has revolutionized cancer treatment, with the anti-PD-1/PD-L1 axis therapy demonstrating significant clinical efficacy across various tumor types. However, it should be noted that this therapy is not universally effective for all PD-L1-positive patients, highlighting the need to expedite research on the second ligand of PD-1, known as Programmed Cell Death Receptor Ligand 2 (PD-L2). As an immune checkpoint molecule, PD-L2 was reported to be associated with patient’s prognosis and plays a pivotal role in cancer cell immune escape. An in-depth understanding of the regulatory process of PD-L2 expression may stratify patients to benefit from anti-PD-1 immunotherapy. Our review focuses on exploring PD-L2 expression in different tumors, its correlation with prognosis, regulatory factors, and the interplay between PD-L2 and tumor treatment, which may provide a notable avenue in developing immune combination therapy and improving the clinical efficacy of anti-PD-1 therapies.

## 1 Introduction

The threat of cancer to human health continues, highlighting the vital importance of early detection, diagnosis and treatment in tumor management. However, the challenge in early diagnosis and treatment arises from the inconspicuous symptoms and lack of specificity in early-stage tumors. The current surgery, chemoradiotherapy, targeted therapy, and combined treatment in a variety of ways have greatly improved the therapeutic effect of tumors, however, due to the severe side effects and drug resistance, their efficacy remains unsatisfactory (1, 2). Hence, there is an imperative to innovate and develop novel treatments that can overcome these limitations. The advent of immunotherapy has revolutionized cancer treatment, owing much of its success to the breakthroughs in immune checkpoint blockade.

Programmed cell death-1(PD-1) is a crucial immunosuppressive molecule with two known ligands, programmed cell death-ligand 1(PD-L1) and PD-L2. The interaction between PD-1 and its ligands results in two opposing effects. One effect is the inhibition of T-cell immunity and the reduction of unnecessary immune responses, contributing to the prevention of autoimmune diseases. Another is the inhibition of the immune system’s ability to monitor and clear tumor cells, thereby allowing the occurrence and development of tumors (3). At present, many studies have been carried out on the PD-1/PD-L1 axis. Certain drugs, including Nivolumab and Pembrolizumab, are employed in the treatment of malignant tumors. However, it has been observed during treatment that not all PD-L1-positive patients exhibit a discernible response to anti-PD-1/PD-L1 axis treatment. Conversely, some PD-L1-negative patients show a positive response to the anti-PD-1/PD-L1 axis treatment, indicating that there may be other molecules or receptors that interact with PD-1 (4).

As the second known ligand of PD-1, whether PD-L2 can be used as another target for tumor therapy has attracted the attention of many researchers. As research progresses, potential interactions and relationships between PD-L1 and PD-L2 emerge. Simultaneous or separate expression of these two ligands can yield varied outcomes in terms of tumor development and prognosis. In a study involving 172 patients with head and neck tumors treated with pembrolizumab, the overall response rate (ORR) was two times higher in both PD-L1 and PD-L2 positive patients than in patients positive for PD-L1 alone. When PD-L2 expression was confined to tumor cells, the ORR was 26.5% in PD-L2 positive patients compared to 16.7% in PD-L2 negative patients (4). These findings indicate a potential relationship between PD-L1 and PD-L2, highlighting the need for investigating anti-PD-1/PD-L2 axis therapy. This may enhance the therapeutic efficacy of tumors when combined with anti-PD-1/PD-L1 axis therapy.

**Figure 1:**
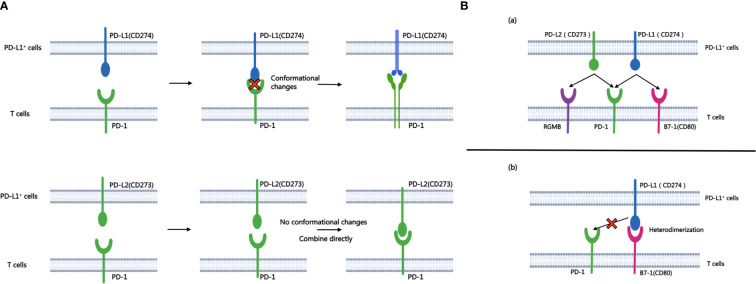
A shows the process of combining PD-L1 and PD-L2 with PD-1 respectively. B.**(A)** Besides PD-1, another binding site of PD-L1 and PD-L2 is PD-L2-RGMB and PD-L1-CD80. **(B)** CD80 can heterodimerize with PD-L1, which prevents the interaction between PD-L1 and PD-1.

The binding affinity of PD-1 with PD-L1 and PD-1 with PD-L2 is quite different. While both ligands exhibit binding affinities to PD-1, structural analyses reveal significant differences in their interaction mechanisms. The binding of PD-1 and PD-L1 induces complex molecular configuration changes(**Figure 1A**), whereas the interaction between PD-1 and PD-L2 is simpler (5). Surface plasmon resonance analyses highlight that PD-L2 demonstrates 2 to 6 times higher affinity for PD-1 compared to PD-L1 (6), and in some cases, the affinity is reported to be 30 times higher (7). PD-L1 and PD-L2 not only bind to PD-1, but also have their own second binding sites. PD-L1 binds to CD80 (8) while PD-L2 binds to RGMb (9). CD80 functions as a co-stimulatory factor during T lymphocyte activation when CD86 activates, playing a pivotal role in autoimmune surveillance, humoral immune response, and transplantation reactions. The interaction between CD80 and PD-L1 results in heterodimerization, thereby impeding the interaction between PD-L1 and PD-1 (**Figure 1B**). However, the interaction of PD-L2 with PD-1 is not negatively regulated by the level of CD80 (10). Therefore, this also explains to some extent that anti-PD-L1 therapy is not effective for all PD-L1 positive patients, which further highlights the necessity and importance of studying PD-L2 and anti-PD-L2 drugs. This heightened affinity has implications for the development of small-molecule compound drugs and the PD-1/PD-L2 axis.

This paper reviews the current status of PD-L2 research in tumors, encompassing the expression of PD-L2 in various common tumors, the association between PD-L2 expression and prognosis, regulatory factors, and the interplay between PD-L2 and tumor treatment.

## 2 Expression of PD-L2 in tumors and its relationship with prognosis

Studies have identified the expression of PD-L2 in various common tumors, including but not limited to lung cancer, colorectal cancer, gastric cancer, esophageal cancer, head and neck cancer, breast cancer, cervical cancer, and liver cancer. High expression of PD-L2 tends to be associated with poor prognosis (**Table 1**).

**Table 1:** The expression of PD-L2 in tumors and its association with clinical prognosis.

Tumor	Expression of PD-L1	Expression of PD-L2	High expression of PD-L2 and Prognosis	Particular relevance	Refs
Lung cancer	Percent high: 59.7%	Percent high:46.3%	Poor prognosis	Present different expression states in different pathological types.	(11, 12)
Head and neck tumor	Percent high: 36.7%	Percent high:36.7%	Poor prognosis Larger tumor size	Expressed in tissues including tumor cells, tumor stromal cells, associated immune cells, lymphoid tissues, and lymph node metastases.	(7–9)
Colorectal cancer	Percent high:36.9%	Percent high: 30.8%	Poor prognosis Pathological grade, vascular invasion, positive surgical margin, LIR and MSI.	Positively correlated with neural infiltration and negatively correlated with CD8 tumor infiltrating cells.	(13, 14)
Gastric cancer	Percent positive: 33% Percent positive in TIIC: 68.0%	Percent positive:28.4% Percent positive in TIIC: 79.9%	Poor prognosis	Highly expressed in at least 20% of neutrophils and predicted a poor prognosis.	(15, 16)
Esophageal cancer	Percent high: 45.5%	Percent high: 59.7%	Poor prognosis	Overexpressed in most esophageal squamous cell carcinomas.	(17, 18)
Breast cancer	Percent positive in BCBM: 53% Positive rate of 56.6%	Percent positive in BCBM:36% Percent positive:50.8%	Poor prognosis	Highly expressed in basal-like, estrogen receptor ER+, HER2-enriched breast cancer, and triple negative breast cancer, as well as widespread in brain metastatic breast cancer	(19–23)
Carcinoma of uterine cervix	Percent positive: 56.0%	Percent positive:53.0%	Poor prognosis	Higher in high-grade CIN patients than in low-grade CIN. Associated with interferon induction.	(24)
Ovarian cancer	Percent high:44.2%	Percent high:22.1%	Poor prognosis	Associated with high CD8, CD68 and other immune molecules.	(25, 26)
Hepatocellular carcinoma	Percent high:27.1%	Percent high: 46.3%	Poor prognosis		(27–30)
Prostatic cancer			Poor prognosis		(31)
Ductal carcinoma of pancreas	No obvious expression	Percent positive:71.5% Percent positive in metastatic tumors: 73%	Poor prognosis	Expressed in most primary and metastatic tumors with reduced immune infiltration. Strong correlation with the density of CD3+, CD8+T cells and FOXP3+regulatory T cells.	(32, 33)
Melanoma	Percent high: 49%	Percent high:37%	Poor prognosis	Highly expressed in patients with decreased immune infiltration.	(34)

(Percent positive: Percentage of samples with PD-L1/PD-L2 mRNA expression detected in tumor cells to total samples. Percent high: The expression of PD-L2 gene was quantitatively determined by quantitative PCR. Compared with other genes (such as housekeeping gene), if the expression of PD-L2 gene is higher than the average level, it is highly expressed. The ratio of high expression samples to total samples is the high expression rate).

## 3 The regulatory network of the PD-L2

### 3.1 Signaling pathways

In tumor cells, the PD-L2 can be regulated by multiple signaling pathways, playing an important role in the occurrence and development of tumors. Therefore, it is imperative to devote sufficient attention to these signaling pathways to enhance our comprehension of the role of PD-L2 in tumorigenesis.

#### 3.1.1 JAK/STAT signaling pathway

The JAK/STAT pathway, formally recognized as the Janus kinase/signal transducer and transcriptional activator signaling pathway, stands as a pivotal communication hub in cellular function. An increasing body of evidence suggests that aberrations in the JAK/STAT pathway are linked to various cancers and autoimmune diseases (35). Studies have reported that the JAK/STAT pathway can trigger the expression of PD-L2 in tumor-related macrophages in lung adenocarcinoma. Additionally, soluble factors derived from cancer cells can induce the overexpression of PD-L2 in macrophages (36), hereby promoting tumor initiation and progression. Overexpression of PD-L1/PD-L2 in some malignant lymphomas is further linked to the activation or abnormalities of the JAK/STAT pathway, often accompanied by alterations in chromosome 9p24.1 (37, 38). These findings substantiate the role of the JAK/STAT pathway in regulating PD-L2 expression.

#### 3.1.2 WNT signaling pathway

WNT (Wingless/Integrated) signaling serves as a crucial molecular regulator guiding a spectrum of physiological processes, encompassing embryonic development, adult stems cell homeostasis, and tissue regeneration (39, 40). Aberrations in the WNT signaling pathway are associated with the development and dysfunction of immune cells, as well as the promotion of immune escape (41). In tumor cells exhibiting high PD-L2 expression, a concomitant reduction in immune infiltration was often observed. In genetic identification, overexpression of genes within the WNT signaling pathway significantly correlated with the lack of immune infiltration (32). In melanoma, PD-L2 expression is also significantly increased in tumor cells with high phosphorylated β-catenin(pS1-βcat protein) expression downstream of the WNT/β-catenin signaling pathway (42), and the strong correlation between the high expression of this downstream protein and PD-L2 may offer insights for future tumor treatments targeting the signaling pathway.

#### 3.1.3 NF-κB signaling pathway

The transcription factor Nuclear factor-κB(NF-κB) plays a pivotal role in inflammation, oncogenesis, and tumor progression, being aberrantly activated in the majority of cancers, thereby contributing to tumorigenesis and progression (43). In a mouse liver cancer model, prolonged indomethacin use was observed to upregulate the expression of PD-L1 and PD-L2 through the TRIF/NF-κB and the JAK/STAT1 pathway. This effect ultimately led to a poor prognosis for hepatocellular carcinoma (HCC) by suppressing Tumor necrosis factor-α(TNF-α) and Interferon gamma-γ(IFN-γ) in liver cancer cells (44). Similarly, an investigation into the pathogenesis of systemic lupus erythematosus identified two crucial pathways, Toll-like receptor and type I interferon, which play a significant role in the development of Systemic lupus erythematosus. These pathways regulate the expression of PD-1 and its ligands (PD-L 2, PD-L1) through the activation of NF-κB, thereby promoting the occurrence and development of systemic lupus erythematosus (45). Furthermore, genetic analysis of clear cell renal carcinoma also revealed an association between the expression of PD-L2 and mRNA levels of NF-κB p65 (46).

#### 3.1.4 AKT/mTOR signaling pathway

Protein kinase B/mammalian target of rapamycin (PI3K/Akt/mTOR) signaling pathway is a fundamental regulator of key physiological processes, including cell growth, migration, survival, and metabolism. Its dysregulation, a prevalent anomaly in tumor patients, significantly contributes to the initiation and progression of tumors (47). In a study focused on gene mutations in meningiomas, heightened PD-L2 expression was observed in patients with mutations in the AKT/mTOR signaling pathway when compared to other immune checkpoint proteins (48). In addition, Irina et al. underscored the pivotal role of PD-L2 in metastatic tumors, emphasizing its significance in tumor progression. They found that the level of the VHL protein, a tumor suppressor gene, determined the invasion capacity of tumors. By strategically targeting VHL through pathological means, they induced changes in the transcriptional and AKT/mTOR regulatory pathways, thereby influencing the expression of PD-L2. This intricate interplay ultimately fuels the initiation and progression of tumors (46, 49).

#### 3.1.5 TLR9 signaling pathway

Known as Toll-like receptor 9, is a key receptor for DNA recognition in cells, activating both innate and acquired immune responses and playing a crucial role in the immune system (50). Ongoing reports and clinical studies suggest promising potential for TLR9 agonists in cancer treatment (51). Baruah et al. reported that high expression of PD-L1/PD-L2 in fibroblasts from HPV-positive head and neck tumor patients is mediated by TLR9, which reduces the expression of PD-L1/PD-L2 when using the TLR9 specific antagonist, ODNTTAGGG (52). This finding opens a new path for targeted TLR9 to regulate immune checkpoint PD-1 and its ligands, which may be of important significance for the treatment of tumors. Moreover, this observation hints at a potential role for HPV in the intricate interplay of TLR9 and immune checkpoint modulation.

### 3.2 Genome and transcription

At the level of genome regulation, the amplification, deletion, or mutation of PD-L2-related genes will change the expression of PD-L2 at the source. The amplification of 9p24.1 will increase the transcription of the PD-L2 gene through Janus kinase 2(JAK2)/signal transduction and transcription activator 1(STAT1) signaling pathway (53). PD-L1-L2-SE, a super-enhancer located between the genes of CD274 and CD273, can induce and activate the transcription of PD-L2 (54). Methylation of the PD-L2 gene reduces the production of PD-L2 protein by inhibiting transcription. Histone acetylation will change the contact between histone and DNA and increase the transcription of the PD-L2 gene. Myc and HOXC10 are transcription-stimulating factors, and combining with the promoter region of PD-L2 gene will significantly increase the transcription level of PD-L2. Ganetespib, located upstream of Myc, suppresses the activity of heat shock protein 90 (HSP90), resulting in an alteration of PD-L2 expression (55). E26 transformation-specific variant transcription factor (ETV4) belongs to the ETS transcription factor family, which enhances PD-L2 transcription by binding to the PD-L1-L2-SE region. When octamer binding protein 2 (OCT2) binds to PD-L2 intron, OCT2 can increase the localization of PD-L2 on the B cell membrane. These regulatory sites may become potential targets for tumor immunotherapy (56).

### 3.3 Non-coding RNA

**Figure 2:**
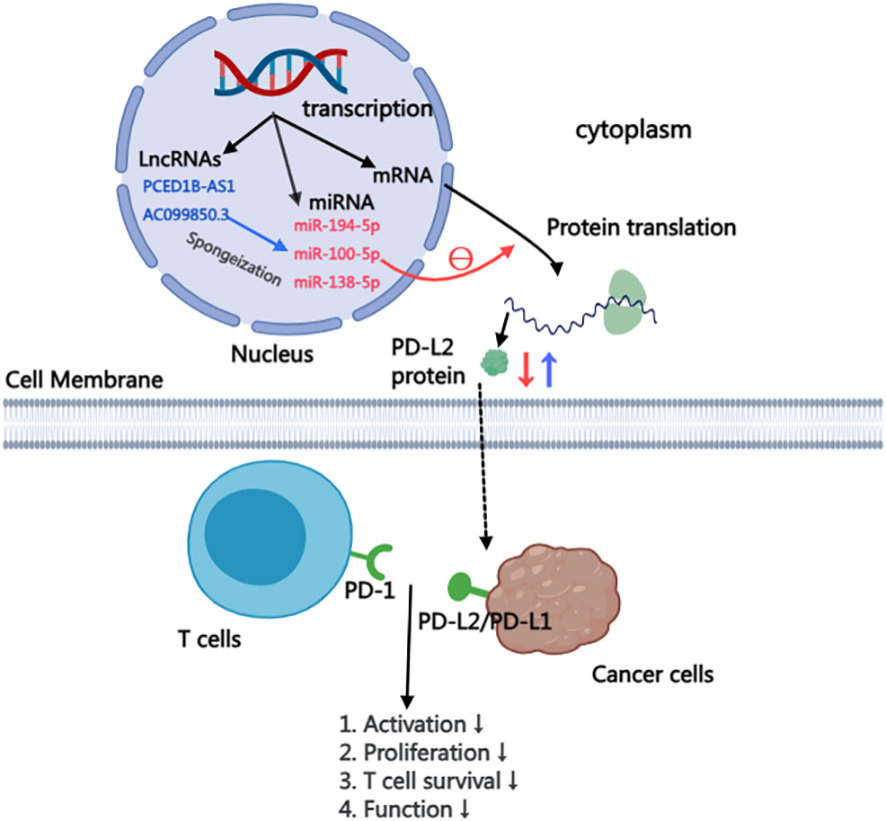
Mechanism of LncRNA and miRNA affecting PD-L2 expression in cells.

The majority of RNA transcribed from human genes cannot encode proteins, constituting what is known as non-coding RNAs. Despite their inability to code for proteins, these non-coding RNAs, including microRNA(miRNA), Long non-coding RNA(LncRNA), intron RNA, and repetitive RNA, exert significant influence on normal gene expression and contribute to various aspects of disease development. This study delves into the intricate impact of non-coding RNAs on the PD-L2, providing insights that highlight their potential as therapeutic targets for anti-tumor drugs (57). within this context, particular emphasis is placed on summarizing and elucidating the nuanced effects of miRNA and lncRNA on PD-L2 (**Figure 2**).

#### 3.3.1 MiRNA

MicroRNA, a subtype of non-coding RNA with a compact length ranging from 19 to 25 nucleotides, exerts its functional impact through post-transcriptional silencing of target genes. Functionally, miRNA plays a pivotal role in post-transcriptional silencing of target genes (58). This discussion succinctly summarizes the current understanding of miRNAs, focusing specifically on their roles in regulating the PD-L2, as detailed in **Table 2**.

**Table 2:** Summary of factors regulating PD-L2.

Regulation type	Action site	Brief mechanism and its influence on PD-L2	Refs
**Signal channel**	JAK/STAT pathway	JAK/STAT pathway induces PD-L2 overexpression in macrophages through soluble factors derived from cancer cells.	(36)
	WNT signaling pathway	Abnormal WNT signaling pathway can induce high expression of PD-L2 and promote immune infiltration deficiency.	(32)
	NF-κB signaling pathway	The expression of PD-L2 gene is related to the mRNA level of NF-κB p65. NF-κB regulates the expression of PD-1 and its ligand PD-L1/PD-L2, which eventually induces poor prognosis of tumor.	(44, 45)
	AKT/mTOR signaling pathway	When gene mutation occurs in AKT/mTOR signaling pathway, the expression of PD-L2 is significantly higher than other immune checkpoint proteins.	(46, 49)
	TLR9 signaling pathway	Toll-like receptor 9 is responsible for activating innate immune cells and mediating the high expression of PD-L1/PD-L2 in fibroblasts, thus promoting tumor progression.	(52)
**Genome and transcription**	9p24.1 gene amplification	The amplification of 9p24.1 will increase the transcription of PD-L2 gene through JAK2/STAT1 signaling pathway.	(53)
	PD-L1–L2-SE	As a super enhancer, PD-L1–L2-SE can induce and activate PD-L2 transcription, which is expected to become a therapeutic target.	(54)
	Myc	The combination of transcription stimulating factor Myc with the promoter region of PD-L2 gene will significantly increase the transcription level of PD-L2.	(59)
	HOXC10	The combination of transcription stimulating factor HOXC10 with the promoter region of PD-L2 gene will significantly increase the transcription level of PD-L2.	(60)
	HSP90	HSP90 is located upstream of Myc, which can significantly down-regulate the expression of PD-L2 by affecting Myc.	(55)
	OCT2	When OCT2 binds to the intron of PD-L2 gene, OCT2 can increase the localization of PD-L2 on B cell membrane and further induce tumor immune escape.	(56)
**Non-coding RNA**	miR-194-5p	miR-194-5p targets the RNA transcript of PD-L1/PD-L2 and inhibits the translation of PD-L1/PD-L2, which is beneficial for the immune system to eliminate tumor cells.	(61)
	miR-100-5p and miR-138-5p	The expression of miR-100-5p was negatively correlated with tumor stage. miR-100-5p inhibits the translation of the corresponding mRNA of PD-L1/PD-L2, and decreases the protein of PD-L1/PD-L2, which is beneficial to the occurrence of tumor immunity.	(62)
		The expression of miR-138-5p was negatively correlated with tumor stage. miR-138-5p inhibits the translation of the corresponding mRNA of PD-L1/PD-L2, and decreases the protein of PD-L1/PD-L2, which is beneficial to the occurrence of tumor immunity.	(62)
	PCED1B-AS1	PCED1B-AS1 regulates the expression of PD-Ls by sponging miR-194-5p.	(61)
	AC099850.3	AC099850.3 is a new type of LncRNA, which acts as an important immune checkpoint for regulating the expression of immune-related genes *in vivo*, and has a significant positive correlation with PD-L1/PD-L2.	(49)
	TLC6	TCL6 has a significant positive correlation with the important immune checkpoints such as PD1, PD-L1 and PD-L2.	(63)
**Enteric microorganisms**	C. Cateniformis	Down-regulating the expression of PD-L2 on antigen presenting cells and the combination of PD-L2 and RGMb to promote anti-tumor immune response.	(64, 65)

*MiR-194-5p:* Plays a crucial role in the onset and advancement of various malignant tumors, encompassing HCC, renal cell carcinoma, melanoma, and pancreatic carcinoma (66–68). Mechanistically, miR-194-5p predominantly targets the RNA transcripts of PD-L1/PD-L2, hindering the translation of PD-L2 mRNA and consequently diminishing the protein content of PD-L2. Experimental evidence presented by Fan et al. empha sizes that hsa-mir-194-5p has the ability to interact simultaneously with the RNA transcripts of both PD-L1 and PD-L2. Through the use of miR-194-5p mi mics and inhibitors, it was observed that inhibiting miR-194-5p led to an increase in the protein levels of PD-L1/PD-L2, while miR-194-5p mimics resulted in a decrease in the protein levels of PD-L1/PD-L2. These findings distinctly indicate that miR-194-5p exerts targeted action on PD-Ls, inhibiting the translation of PD-1 ligand proteins. Consequently, in cases of abnormal function or absence of miR-194-5p, an overproduction of PD-L2 protein occurs. The interplay between PD-1 and PD-L2 induces immunosuppression in hepatocellular carcinoma, thereby fostering the initiation and progression of the tumor (61).

*MiR-100-5p and miR-138-5p:* Studies have shown that miR-100-5p and miR-138-5p are related to mitogen-activated protein kinase (MAPK) signaling pathway, apoptosis, and tumor necrosis factor (TNF) pathway, all of which are the regulators of cancer development, progression, and immune escape (62). In a study conducted by EI Ahanidi et al., the investigation into the relationship between miR-100-5p, miR-138-5p, and the TERT/PDL1/PD-L2 axis demonstrated that miR-138-5p exhibited elevated expression in normal bladder tissue, displaying a consistent downward trend from low-grade to high-grade tumors and from early-stage to progressive tumors. Notably, both miRNAs showed a negative correlation with tumor staging in bladder cell lines, and this negative correlation extended to the expression of PD-L1/PD-L2 protein (62). These results indicated that the combination of miR-100-5p and miR-138-5p to target genes inhibited their mRNA translation, resulting in the decrease of the expression level of PD-L1/PD-L2 protein, and finally induced immunosuppression of the tumor, further promoting the malignant progression and poor prognosis of the tumor.

#### 3.3.2 LncRNA

Long non-coding RNA, defined by a length exceeding 200 nucleotides, plays a pivotal role in diverse biological processes. As a central focus in genetic research, LncRNA has been increasingly recognized as a critical regulatory factor for protein-encoding genes in various diseases, particularly cancer, where it significantly contributes to both the initiation and progression of tumor (69). Recent studies delving into the intricate network between lncRNA, and microRNA have revealed their regulatory role in immune checkpoint gene expression in breast cancer. This suggests that LncRNA may influence tumor progression by modulating immune checkpoint pathways (70).

*PCED1B-AS1:* Has been implicated in the pathogenesis of various human cancers, including gastric cancer, colorectal cancer, pancreatic ductal cancer, and renal clear cell carcinoma (71–74). Recent investigations highlight the role of lncRNA in regulating multiple target genes by acting as a miRNA sponge, effectively inhibiting miRNA function. Given the crucial role of PD-L1/PD-L2 in mediating tumor immunosuppression, Fan et al. reported that the long-chain non-coding RNA PCED1B-AS1 may function as a cytoplasmic sponge, modulating the expression of PD-Ls by sequestering miR-194-5p. Knocking down PCED1B-AS1 using lentivirus shRNA in a liver cancer cell line resulted in a simultaneous reduction in the protein levels of PD-L1 and PD-L2. Conversely, lentivirus overexpression of PCED1B-AS1 increased the protein levels of PD-L1 and PD-L2. These findings suggest that PCED1B-AS1 exerts control over the expression of PD-L1/PD-L2 in hepatoma cells by inhibiting miRNA function. Aberrations in PCED1B-AS1 may induce immunosuppression in hepatocellular carcinoma, ultimately contributing to tumor deterioration and progression (61).

*AC099850.3:* lncRNA-AC099850.3 emerges as a novel player with abnormal expression across various tumor types, including lung adenocarcinoma and hepatocellular carcinoma, showing associations with tumor staging, poor prognosis, and immune infiltration (75, 76). In HCC, the knockdown of AC099850.3 significantly impairs the *in vitro* proliferation and invasion capabilities of HCC and inhibits tumor cell growth *in vivo*. Notably, AC099850.3 exhibits a marked positive correlation with PD-L1 and PD-L2, key immune checkpoints *in vivo*. High expression of PD-L1/PD-L2 is generally linked to poor tumor prognosis, indicating that AC099850.3 functions as an immune-related gene, regulating the expression of critical immune checkpoints in HCC. This, in turn, induces immunosuppression in HCC. Furthermore, studies have revealed that AC099850.3 promotes the malignant progression of hepatocellular carcinoma through the AKT signaling pathway. Spirina et al. reported that the AKT signaling pathway exerts a regulatory effect on PD-L2 (49).

*TLC6:* T-cell leukemia/lymphoma 6, a novel regulator implicated in HCC and renal clear cell carcinoma progression, associated with a poor prognosis (77, 78). In breast cancer, low TCL6 expression independently predicts an adverse outcome for PR (estrogen and progesterone receptor)-negative patients, while also correlating with immune infiltrating cells, including B cells, CD4+ T cells, and CD8+ T cells. Furthermore, TCL6 shows a significant positive correlation with PD1, PD-L1, and PD-L2 (63). However, the pathway or mechanism by which TCL6 acts on the expression of PD-L1/PD-L2 still needs further study (20). TCL6 presents a promising avenue for exploration in cancer therapeutics.

### 3.4 Gut microbiome

A large number of studies have shown that microorganisms in the intestinal tract and other parts may promote the occurrence and development of cancer, and affect cancer immune surveillance and response to immunotherapy (79). Studies have reported that intestinal microflora can down-regulate the expression of PD-L2 in CD11c+DCs (dendritic cells) in the intestine and tumor drainage lymph nodes and can promote anti-tumor immunity by combining with partner repulsion guiding molecule B (RGMB). Park et al. discovered an intestinal flora-C. cateniformis, which can induce the above-mentioned effects. The combination of PD-L2 and RGMB plays a role in inhibiting anti-cancer T cells. Blocking the interaction between PD-L2 and RGMB with antibodies can further relieve the inhibition of T cells, which is beneficial for T cells to clear cancer cells. This indicates that PD-L2 not only inhibits T cells through PD-1 but also exerts inhibitory effects through RGMB (64, 65). This also confirmed a new strategy of cancer immunotherapy-blocking the interaction between PD-L2 and RGMB to enhance the response to cancer immunotherapy.

## 4 Treatment strategy

### 4.1 Small molecular drugs

Because the affinity of PD-1 to PD-L1 and PD-1 to PD-L2 is quite different. These small molecules hold the potential to penetrate the tumor microenvironment more effectively than traditional anti-PD-1/PD-L1 axis drugs, such as pabolizumab, potentially enhancing therapeutic efficacy (80). One notable molecular targeted drug in this context is JQ1, a BET-bromine domain inhibitor. JQ1 inhibits the binding of the BET-bromine domain and histone, suppressing the transcription of target genes. In studies conducted by Liu et al., JQ1 treatment led to a reduction in PD-L2 mRNA levels in renal cell carcinoma, and prostate, liver, and lung cancer cell lines (81). Therefore, JQ1 may be a potential molecular drug for tumor treatment. **Table 3** summarizes the current new tumor treatment strategies for PD-L2. Although some of them are still in the research stage, they represent the new direction of tumor treatment in the future.

**Table 3:** Tumor treatment strategy for PD-L2.

Therapeutic strategy	Mechanism of action	Refs
**Small molecule drug**	Because the binding affinity of PD-L2 and PD-1 is much higher than that of PD1-PD-L1, small molecule targeted drugs can penetrate into tumor microenvironment more effectively, thus enhancing the therapeutic effect. Such as JQ1.	(81, 82)
**Monoclonal antibody**	Different monoclonal antibodies act at different stages. (a) acting on the upstream of PD-L2 can reduce the expression of PD-L2, such as Ganetespib; (b) Acting on the junction of PD-L2 and PD-1 can directly inhibit the function of PD-L2, such as Dostarlimab; (c) It can also be applied to PD-L2-RGMb. All these can inhibit the immune escape of tumor.	(83, 84)
**PD-L2 vaccine**	Relying on PD-L2-specific T cells naturally produced by human body, these T cells can directly kill PD-L2-positive target cells, thus inhibiting PD-L2-mediated immune escape.	(85)
**Flora transplantation**	Gram-positive anaerobic bacteria in human intestinal flora can promote anti-tumor immune response by down-regulating the expression of PD-L2 and combining RGMB to some extent.	(65)
**Anti-PD-L2 combined Radiotherapy**	Radiotherapy radiation will activate the immune system, and the expression of PD-L2/PD-L1 will increase in some tumor patients, so radiotherapy combined with anti-PD-L1/PD-L2 therapy will improve the tumor treatment effect to some extent.	(86–88)

### 4.2 PD-L2 vaccine

The so-called PD-L2 vaccine is to introduce PD-L2 antigen or proteins or cells carrying the restricted epitopes of human leukocyte antigen (HLA) expressed by PD-L2 into patients, thus inducing the activation of PD-L2 specific T cells. PD-L2-specific T cells, naturally produced in cancer patients, play a dual role in supporting anti-tumor immunity. These T cells can directly kill target cells and indirectly contribute to anti-tumor immunity by releasing pro-inflammatory factors in the tumor microenvironment. In clinical trials, researchers aim to enhance the immune response against PD-L2 by administering the PD-L2 vaccine, hoping to complement other forms of immunotherapy. While the treatment’s effectiveness hasn’t shown significant improvement in patients, it holds potential as a target for cancer vaccine and immune checkpoint-blocking treatment*s* (85).

### 4.3 Combination with radiotherapy

As a vital component of tumor treatment, the potential of radiotherapy interactions with immune checkpoint inhibitors are also reported. A study revealed a significant increase in PD-L1 and PD-L2 expression in MCF-120 (human breast cancer cells) after hyperthermia (HT) and radiotherapy RT (2×5 Gy) treatment at any temperature (86). Considering the elevated expression of immune checkpoint molecules, incorporating immune checkpoint inhibitors into the combined tumor hyperthermia and radiotherapy approach may optimize therapeutic outcomes. In addition, radiotherapy is previously reported to be able to locally activate the immune system, potentially inducing a systemic immune response and targeting distant metastatic tumors (87). Previous research demonstrated increased efficacy of paolizumab in lung cancer patients who underwent prioradiotherapy (88). And the activation of immune system by radiotherapy radiation may be optimized and improved in tumors with more inhibition of immune system by PD-L2 at baseline. However, understanding the pathway through which radiotherapy affects PD-L1/PD-L2 and investigating the potential relationship between the immune response induced by local radiotherapy require further exploration (31).

### 4.4 Intestinal flora transplantation

Previous studies have shown that transplantation of fecal flora from patients with good response to PD-1 therapy can reduce the drug resistance of melanoma patients to PD-1 therapy, thus improving the tumor treatment effect. Gram-positive anaerobic bacteria in human intestinal microorganisms can promote anti-tumor immune response by down-regulating the expression of PD-L2 and binding RGMB to some extent (65). This will further expand the starting point of tumor treatment. On the one hand, it inspires us to fully strengthen the study of intestinal flora, clarify the types of intestinal microorganisms for tumor treatment, and bring a new dawn to tumor patients who have poor responses to immunotherapy through targeted flora transplantation. On the other hand, using intestinal microorganisms as a discovery platform to identify a new target of cancer immunotherapy-PD-L2-RGMB, and blocking the interaction between PD-L2 and RGMB can enhance the response to cancer immunotherapy. Small molecule drugs targeting specific intestinal microorganisms can be further developed to enhance the effect of cancer immunotherapy.

## 5 Prospects

In conclusion, PD-L2 stands as a key player in various human cancers, showing significant promise as a therapeutic target. Currently, there exist numerous constraints, notably the incomplete comprehension of PD-L2 expression and its regulatory mechanism, as well as the insufficient exploration of related signal pathways. Moreover, the clinical approval of anti-PD-L2 antibodies for tumor treatment remains elusive, necessitating further investigation and discovery. However, the high affinity of PD-L2 for PD-1, the new insight that intestinal microorganisms regulate PD-1 pathway, and the richness of PD-L2 related therapies all indicate that cancer immunotherapy is expected to make a breakthrough. The discovery of new pathways such as PD-L2-RGMB has further expanded the scope of targeted therapy. The continuous exploration of clinical application of PD-L2 has brought exciting prospects for promoting cancer treatment. The full names of abbreviations in this article are displayed in **Table 4**.

**Table 4:** Summary of abbreviations.

Abbreviations	
PD-1	Programmed death-1
PD-L1	Programmed cell death-ligand 1
PD-L2	Programmed cell death-ligand 2
miRNA	microRNA
LncRNA	Long non-coding RNA
ORR	Overall response rate
CIN	Cervical intraepithelial neoplasia
JAK/STAT	Janus kinase/Signal transducer and transcriptional activator
WNT	Wingless/Integrated
NF-κB	Nuclear factor-κB
AKT/mTOR	Protein kinase B/mammalian target of rapamycin
TLR9	Toll-like receptor 9
TNF-α	Tumor necrosis factor-α
IFN-γ	Interferon gamma-γ
TLC6	T-cell leukemia/lymphoma 6
pS1-βcat	phosphorylated β-catenin
MCF-120	Human breast cancer cells
JAK2/STAT1	Janus kinase 2/signal transduction and transcription activator 1
HSP90	Heat shock protein 90
ETV4	E26 transformation-specific variant transcription factor
OCT2	Octamer binding protein 2
TIIC	Tumor immune infiltrating cells

## Author contributions

YY: Writing – original draft, Writing – review & editing. XY: Writing – original draft, Writing – review & editing. XB: Conceptualization, Writing – review & editing. JY: Investigation, Writing – review & editing. JS: Writing – original draft, Writing – review & editing.
